# Molecular properties and In silico bioactivity evaluation of (4-fluorophenyl)[5)-3-phen-(4-nitrophenyl yl-4,5-dihydro-1*H*-pyrazol-1-yl]methanone derivatives: DFT and molecular docking approaches

**DOI:** 10.1016/j.jtumed.2023.05.011

**Published:** 2023-05-23

**Authors:** Ibrahim A. Omotayo, Semire Banjo, Oladuji T. Emmanuel, Latona D. Felix, Oyebamiji A. Kolawole, Owonikoko A. Dele, Abdulsalami I. Olasegun, Adeoye M. Dasola, Odunola O. Ayobami

**Affiliations:** a Computational Chemistry Laboratory, Department of Pure and Applied Chemistry, Ladoke Akintola University of Technology, Ogbomoso, Nigeria; b Department of Pure and Applied Chemistry, Osun State University, Osogbo, Nigeria; c Department of Chemistry and Industrial Chemistry, Bowen University, Iwo, Nigeria; d Department of Chemistry, Emmanuel Alayande College of Education, Nigeria; e Department of Chemistry, Nigerian Army University, Biu, Nigeria; f Department of Chemical Sciences, Fountain University, Osogbo, Nigeria; g Department of Chemistry, Faculty of Natural and Applied Sciences, Hallmark University, Ijebu-Itele, Nigeria

**Keywords:** ميثانون (4-فلوروفينيل) [5- (4-نيتروفينيل) -3-فينيل-4،5-ثنائي هيدرو-1اتش-بيرازول-1-يل], نظرية الكثافة الوظيفية, إرساء جزيئي, DFT, Molecular docking, [5-(4-nitrophenyl)-3-phenyl-4,5-dihydro-1*H*-pyrazol-1-yl]methanone

## Abstract

**Objectives:**

Molecular structures, spectroscopic properties, charge distributions, frontier orbital energies, nonlinear optical (NLO) properties and molecular docking simulations were analyzed to examine the bio-usefulness of a series of (4-fluorophenyl)[5-(4-nitrophenyl)-3-phenyl-4,5-dihydro-1H-pyrazol-1-yl]methanone derivatives.

**Methods:**

The compounds were studied through computational methods. Equilibrium optimization of the compounds was performed at the B3LYP/6-31G(d,p) level of theory, and geometric parameters, frequency vibration, UV–vis spectroscopy and reactivity properties were predicted on the basis of density functional theory (DFT) calculations.

**Results:**

The energy gap (ΔEg), electron donating/accepting power (***ω***−/***ω***+) and electron density response toward electrophiles/nucleophiles calculated for **M1** and **M2** revealed the importance of substituent positioning on compound chemical behavior. In addition, ***ω***−/***ω***+ and ΔEn/ΔEe indicated that **M6** is more electrophilic because of the presence of two NO_2_ groups, which enhanced its NLO properties. The hyperpolarizability (β_0_) of the compounds ranged from 5.21 × 10^−30^ to 7.26 × 10^−30^ esu and was greater than that of urea; thus, **M1–M6** were considered possible candidates for NLO applications. Docking simulation was also performed on the studied compounds and targets (PDB ID: 5ADH and 1RO6**),** and the calculated binding affinity and non-bonding interactions are reported.

**Conclusion:**

The calculated ω^−^ and ω^+^ indicated the electrophilic nature of the compounds; **M6**, a compound with two NO_2_ groups, showed enhanced effects. Molecular electrostatic potential (MEP) analysis indicated that amide and nitro groups on the compounds were centers of electrophilic attacks. The magnitude of the molecular hyperpolarizability suggested that the entire compound had good NLO properties and therefore could be explored as a candidate NLO material. The docking results indicated that these compounds have excellent antioxidant and anti-inflammatory properties.

## المخلص

تمت دراسة سلسلة من مشتقات الميثانون (4-فلوروفينيل) [5- (4-نيتروفينيل) -3-فينيل-4،5-ثنائي هيدرو-1اتش-بيرازول-1-يل] باستخدام الطرق الحسابية. تم إجراء تحسين التوازن لهذه المركبات عند مستوى "ب3ليب/6-31ج∗∗" من الناحية النظرية، وتم التنبؤ بالمعلمات الهندسية، والاهتزاز الترددي، وخصائص الأشعة المرئية وفوق البنفسجية على أساس حسابات نظرية الكثافة الوظيفية. كشفت فجوة الطاقة، والتبرع بالإلكترون / قبول الطاقة واستجابة كثافة الإلكترون تجاه الإلكتروفيل / النوكليوفيل المحسوبة لـ إم1 و إم2، عن أهمية وضع البديل على السلوك الكيميائي للمركب. أيضا، أظهر التبرع بالإلكترون / قبول الطاقة و فجوة الطاقة أن إم6 أكثر إلكتروفيلا بسبب وجود مجموعتين من "إن أو 2"، مما يعزز خصائص "إن إل أو" الخاصة به. تراوحت قابلية الاستقطاب المفرط للمركبات 5.21-7.26 × 10^-30^ وحدة كهرباء وكانت أكبر من مادة اليوريا القياسية "إن إل أو"؛ وبالتالي جعل إم1-إم6 مرشحين محتملين لتطبيقات "إن إل أو". كشفت عمليات محاكاة الإرساء أن التقاربات الملزمة للمركبات الستة تراوحت من -8.8 إلى -9.3 كيلو كالوري / مول لـ "أ ب أو"-نازعة هيدروجين الكبد (عنوان بي دي بي: 5 أ دي إتش) و -8.5 إلى -9.7 كيلو كالوري / مول لـ هيدرولاز البروتين المضاد للالتهابات (عنوان بي دي بي: 1 آر أو 6)؛ وبالتالي يمكن أن تمتلك خصائص جيدة مضادة للأكسدة ومضادة للالتهابات.

## Introduction

Pyrazole derivatives are heterocyclic compounds recognized to possess a wide range of biological and pharmacological activities.^1^^,^^2^ The unique properties of pyrazoles have been attributed to the electrophilic substitution reactions that occur specially at position 4, and nucleophilic attacks that occur at positions 3 and 5, thus leading to diverse pyrazole structures with broad potential applications in areas such as medicine, agriculture and technology.^3^, ^4^, ^5^ In addition, compounds containing a pyrazole nucleus have been reported to be anti-inflammatory, antiviral, anticancer, antiparasitic, antibacterial, antirheumatoid, antidepressant, analgesic, antinociceptive, antihypertensive, antipyretic and antifungal agents.^6^, ^7^, ^8^, ^9^, ^10^, ^11^, ^12^ Beyond these biological activities, this class of compounds displays substantial nonlinear optical (NLO) properties,^13^, ^14^, ^15^, ^16^, ^17^ electroluminescent properties due to photo-induced-electron transfer,^18^ and light amplification properties due to stimulated emission or lasing/random lasing action.^14^^,^^15^

Several pyrazole derivatives, such as 3-(1,1-dicyanoethenyl)-1-phenyl-4,5-dihydro-1*H*-pyrazole, have been investigated for their NLO properties. The size of the nano-crystals of the compound has been suggested to play a major role in the excitation or emission efficiency.^19^^,^^20^ Thus, these compounds can be used for ultrafast optics.^21^ The optical nonlinearity of a series of *N*-substituted-5-phenyl-1*H*-pyrazole-4-ethyl carboxylates of compounds in chloroform solution has been assessed, and these compounds have been found to be good candidates for NLO applications.^22^ In addition, a series of (*Z*)-2-(4-nitrophenyl)-3-(1-phenyl-4,5-dihydro-1*H*-pyrazol-3-yl)acrylonitrile and (*E*)-3-(4-nitrostyryl)-1-phenyl-4,5-dihydro-1*H*-pyrazole compounds have been found to have several electron accepting groups attached and to show high NLO responses dependent on functionalization of the pyrazoline derivatives.^23^ The compounds 1-*N*-phenyl-3(3,4-dichlorophenyl)-5-phenyl-2-pyrazoline,^24^ diethyl-1*H*-pyrazole-3,5-dicarboxylate and 4-(4-bromophenyl)-1-tert-butyl-3-methyl-1*H*-pyrazol-5-amine^25^ have been studied with experimental and density functional theory (DFT) methods and found to have promising NLO properties.

More recently, DFT has been used in conformational and NBO analysis of (4-chloro-3,5-dimethyl-1*H*-pyrazol-1-yl) (p-tolyl)methanone, and the results have shown excellent agreement with experimental data.^26^ Likewise, 2-bromo-*N*-(2,3-dihydro-1,5-dimethyl-3-oxo-2-phenyl-1*H*-pyrazol-4-yl)benzamide and 2-chloro-*N*-(2,3-dihydro-1,5-dimethyl-3-oxo-2-phenyl-1*H*-pyrazol-4-yl)benzamide have been studied with both experimental and DFT methods; the theoretical results have revealed that both compounds display energetic hydrogen bonding interactions and are stabilized by electrostatic energy contribution, in line with experimental observations.^27^ Santhi and Bharathi have reported the synthesis and molecular structural elucidation of a series of 4-(3-(2-amino-3,5-dibromophenyl)-1-(benzoyl)-4,5-dihydro-1*H*-pyrazol-5-yl)benzonitriles through spectroscopic and DFT methods. The DFT method has been used to analyze the studied molecules’ molecular electrostatic potential, natural bonding orbitals, Mulliken charges, frontier molecular orbital energies and NLO properties. The results have indicated inter- and intra-molecular delocalization and acceptor–donor interactions based on second-order perturbation interactions; moreover, polarizability and hyperpolarizability calculations have indicated that the compounds possess good NLO properties.^28^

In this work, DFT calculations were performed on 4-(3-(2-amino-3,5-dibromophenyl)-1-(benzoyl)-4,5-dihydro-1*H*-pyrazol-5-yl)benzonitriles reported by Santhi and Bharathi.^28^ Structural modifications were used to design six new compounds, as shown in Table 1. The molecular structures, spectroscopic properties, charge distributions, frontier orbital energies, NLO properties and molecular docking simulations were analyzed to examine the bio-usefulness of the studied compounds.

**Table 1:** Schematic structures of the modeled compounds; Ma∗ = compound from.^28^


Compound	R1	R2	Name
**M1**	F	NO_2_	[3-(2-aminophenyl)-5-(4-nitrophenyl)-4,5-dihydro-1*H*-pyrazol-1-yl](4-fluorophenyl)methanone
**M2**	NO_2_	F	[3-(2-aminophenyl)-5-(4-fluorophenyl)-4,5-dihydro-1*H*-pyrazol-1-yl](4-nitrophenyl)methanone
**M3**	CH_3_	NO_2_	[3-(2-aminophenyl)-5-(4-nitrophenyl)-4,5-dihydro-1*H*-pyrazol-1-yl](4-methylphenyl)methanone
**M4**	OH	NO_2_	[3-(2-aminophenyl)-5-(4-nitrophenyl)-4,5-dihydro-1*H*-pyrazol-1-yl](4-hydroxyphenyl)methanone
**M5**	OCH_3_	NO_2_	[3-(2-aminophenyl)-5-(4-nitrophenyl)-4,5-dihydro-1*H*-pyrazol-1-yl](4-methoxyphenyl)methanone
**M6**	NO_2_	NO_2_	[3-(2-aminophenyl)-5-(4-nitrophenyl)-4,5-dihydro-1*H*-pyrazol-1-yl](4-nitrophenyl)methanone
**Ma**	F	CN	4-[3-(2-amino-3,5-dibromophenyl)-1-(4-fluorobenzoyl)-4,5-dihydro-1*H*-pyrazol-5-yl]benzonitrile

## Theoretical details

Before DFT calculations, an equilibrium conformer search was performed on all compounds with a semi-empirical AM1 method to identify the lowest conformer for each compound, which was used for further DFT calculations.^29^ All calculations were performed on these compounds with Becke's three parameter hybrid functional DFT, with Lee, Yang and Parr correlation,^30^ and optimized at B3LYP/6-31G(d,p) level of theory in gas. Frequency calculation was also performed by using the same basis set to confirm that the optimized molecules were minima, as characterized by positive harmonic frequencies^31^^,^^32^ in Spartan 14.^33^ The DFT hybrid B3LYP functional has been reported to overestimate the fundamental modes; however, this overestimation can be addressed by calculating harmonic frequencies with a scaling factor of 0.9619 to yield frequencies consistent with experimental data.^34^ The molecular descriptors calculated from conceptual DFT were the ionization potential (I = −HOMO), electron affinity (A = −LUMO), chemical hardness (η), chemical potential (μ), global electrophilicity (ω), electron donating power $\left(\omega^{-}\right)$, electron accepting power ($\omega^{+})$, nucleofugality (ΔE_n_) and electrofugality (ΔE_e_) (equations 1, 2, 3, 4, 5, 6, 7)).^35^, ^36^, ^37^, ^38^, ^39^

1 $$\mu =\frac{-E_{LUMO}+E_{HOMO}}{2}\approx \frac{I+A}{2}$$

2 $$\mu =-\frac{E_{HOMO}+E_{LUMO}}{2}\approx \frac{I+A}{2}$$

3 $$\omega =\frac{\mu^{2}}{2\eta }=\frac{{\left(I+A\right)}^{2}}{4\left(I-A\right)}$$

4 $$\omega^{-}=\frac{{\left(3I+A\right)}^{2}}{16\left(I-A\right)}$$

5 $$\omega^{+}=\frac{{\left(I+3A\right)}^{2}}{16\left(I-A\right)}$$

6 $$\Delta E_{n}=-A+\omega =\frac{{\left(\mu +\eta \right)}^{2}}{2\eta }$$

7 $$\Delta E_{e}=I+\omega =\frac{{\left(\mu -\eta \right)}^{2}}{2\eta }$$

## Results and discussion

### Geometry parameters

The geometry parameters extracted from the equilibrium structures optimized at the B3LYP/6-31G(d,p) level for the six compounds **M1–M6** are displayed in Tables 2 and 3. The results were compared with the geometries of 4-(3-(2-amino-3,5-dibromophenyl)-1-(4-nitrobenzoyl)-4,5-dihydro-1*H*-pyrazol-5-yl)benzonitrile predicted at the same level of theory.^28^ The calculated C3–N7 bonds were 1.299, 1.298, 1.300, 1.299, 1.299 and 1.298 Å for **M1–M6**, respectively, in agreement with the 1.299 Å calculated for **Ma**. This type of bond has been experimentally observed to be 1.275 Å and calculated to be 1.287 Å for bis-spiropipridinon/pyrazole derivatives.^40^ The C1–N8 bond in the pyrazole ring displayed a typical single bond character with a bond length of 1.482 Å for **M1**, 1.490 Å for **M2**, 1.490 Å for **M3**, 1.479 Å for **M4**, 1.481 Å for **M5,** 1.486 Å for **M6** and 1.487 Å for **Ma**. The N–N bond length in the pyrazole ring was calculated to be 1.376 Å for M1, 1.378 Å for M2, 1.372 Å for **M3**, 1.376 Å for **M4**, 1.375 Å for **M5**, 1.381 Å for **M6** and 1.374 Å for **Ma**; these values have been experimentally found to be 1.385 and 1.369 Å,^40^ and 1.3827 Å^27^ for similar compounds. In addition, The N8–C34 bond length value was 1.383 Å for **M1**, 1.378 Å for **M2**, 1.381 Å for **M3**, 1.360 Å for **M4**, 1.380 Å for **M5**, 1.380 Å for **M6** and 1.374 Å for **Ma**.

**Table 2:** Optimized structures of **M1–M6 in** 2-D and 3-D.

Compound	2-D structure	3-D structure
**M1**		
**M2**		
**M3**		
**M4**		
**M5**		
**M6**

**Table 3:** Selected bond length, bond angle and dihedral angle for the studied compounds.

Bond length(Å)	**M1**	**M2**	**M3**	**M4**	**M5**	**M6**	**Ma**
C1–C2	1.552	1.550	1.547	1.551	1.552	1.550	1.546
C1–N8	1.482	1.490	1.490	1.479	1.481	1.486	1.487
C2–C3	1.523	1.520	1.522	1.522	1.523	1.522	1.522
C3–N7	1.299	1.298	1.300	1.299	1.299	1.298	1.295
N7–N8	1.376	1.378	1.372	1.376	1.375	1.381	1.374
C3–C9	1.457	1.458	1.457	1.450	1.457	1.457	1.457
N8–C34	1.383	1.378	1.381	1.360	1.386	1.380	1.381
C34–O35	1.228	1.228	1.235	1.221	1.229	1.227	1.223
C11–N17	1.358	1.357	1.36	1.359	1.357	1.357	1.358
C14–Br20	1.92	1.919	1.92	1.924	1.921	1.919	1.921
C12–Br21	1.916	1.917	1.914	1.914	1.918	1.913	1.914
C29–C32	1.471	1.348	1.466	1.471	1.471	1.472	1.430
C32–N33	1.231	–	1.231	1.231	1.231	1.230	1.155
C43-X46	1.346	1.474	1.498	1.361	1.359	1.474	1.389
Bond angle (°)							
C1–C2–C3	102.65	103.26	102.71	102.41	102.56	103.11	102.80
C2–C3–N7	112.22	112.6	112.23	112.17	112.15	112.63	112.15
C3–N7–N8	109.66	109.65	109.48	109.55	109.74	109.67	109.73
N8–C34–C36	119.93	119.14	102.67	120.37	119.1	119.2	120.22
Dihedral angle (°)							
C11–C9–C3–N7	−0.64	0.30	−2.42	−3.53	−0.72	1.85	−2.94
C10–C9–C3–C2	0.30	0.35	−0.77	−2.30	0.26	1.91	−2.09
C2–C1–C22–C24	111.01	−52.34	−41.6	−71.79	−110.87	−57.52	−73.11
N8–C1–C22–C23	47.07	62.37	−92.22	−140.77	46.92	−123.25	−112.47
C1–N8–C34–035	−1.37	1.16	−0.61	−3.23	−1.30	−0.47	−2.35
C38–C36–C34–O35	−30.08	30.81	−10.79	−25.60	−29.55	29.72	−29.56

Ma = Experimental data of the compound from Ref.^28^.

The C2–C3–N7 bond angle for the six compounds was calculated to be 112.22°, 112.6°, 112.23°, 112.17°, 112.15° and 112.63° for **M1–M6**, respectively. However, N7–N8–C3 was 109.66° for **M1**, 109.65° for **M2**, 109.48° for C3, 109.55° for **M4**, 109.74° for **M5** and 109.67° for **M6**. These two bond angles indicated that the pyrazole ring was distorted from planarity by the aryl and benzoyl rings, as reflected in the dihedral angles (Table 3), thus revealing that aryl and benzoyl groups have more profound effects on the pyrazole ring than the extended R1 and R2 substituents.

### Frontier molecular orbital and UV–vis absorption properties

**Figure 1:**
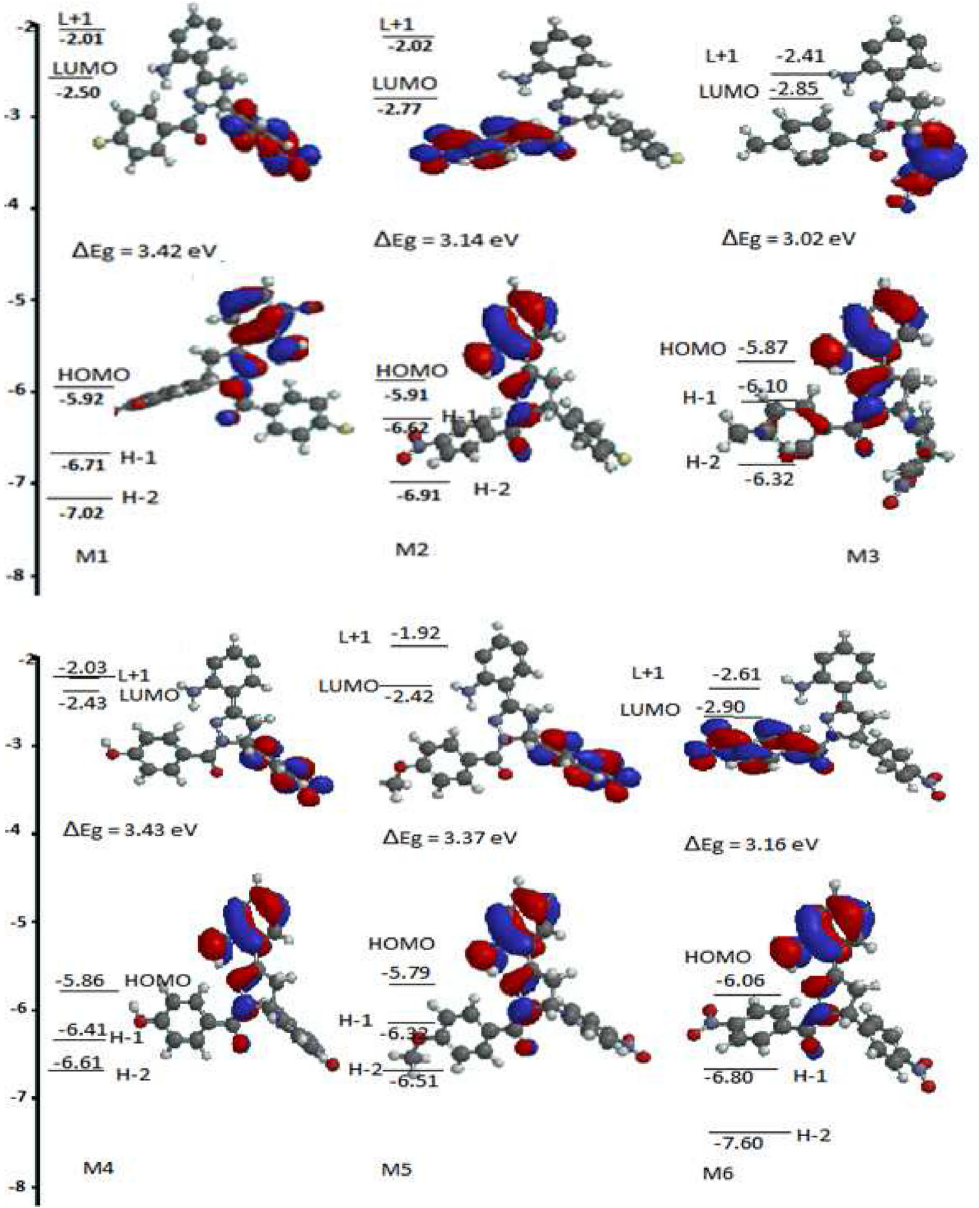
Frontier molecular orbitals for compounds **M1–M6**.

The frontier orbital energies, such as the highest occupied molecular orbital energy (HOMO), lowest unoccupied molecular orbital (LUMO) and band gaps, are critical parameters for kinetic and thermodynamic stability studies, and prediction of reactivity and photochemical properties.^41^, ^42^, ^43^, ^44^, ^45^, ^46^ The frontier orbital molecular overlay revealed that the HOMO overlay was essentially on ring, extending over the two nitrogen atoms of the pyrazole ring, whereas the LUMO overlay was on the phenyl ring for **M1, M3, M4** and **M5**, and was on the benzoyl ring for **M1** and **M6** (Figure 1). The calculated frontier molecular orbitals LUMO, HOMO and HOMO–LUMO (ΔEg) were, respectively, −2.50, −5.92 and 3.42 eV for **M1**; −2.77, −5.91 and 3.14 eV for **M2**; −2.85, −5.87 and 3.02 eV for **M3**; −2.43, −5.86 and 3.43 eV for **M4**; −2.42, −5.86 and 3.37 eV for **M5**; −2.90, −6.06 and 3.16 eV for **M6**; and −2.26, −6.10 and 3.83 for **Ma**. The replacement of a cyano group in **Ma** with NO_2_ as in **M1** led to a decrease in LUMO energy with an increase in HOMO energy, thus resulting in a decrease in ΔEg by 0.41 eV and profound effects on the electron withdrawing capacity of NO_2_ compared with the CN group. The interchanged **M1** F and NO_2_ positions in **M2** further decreased the ΔEg by 0.28 eV with respect to **M1**, thus indicating that the amide group enhanced the electron withdrawing ability of NO_2_. **M3, M4, M5** and **M6** were modeled by the replacement of fluorine in **M1** with CH_3_, OH, OCH_3_ and NO_2_, respectively. **M3, M5** and **M6** presented ΔEg than **M1**; however, **M6** showed the lowest ΔEg because of lowering of the LUMO energy (−2.90 eV) and stabilization of the HOMO, thereby decreasing the π-electron density of the aromatic rings. Thus, **M6** was expected to be relatively more reactive toward nucleophiles (Table 2).

Other calculated reactivity descriptors such as chemical hardness (η), chemical potential (μ) and global electrophilicity (ω) were, respectively, 1.71, −4.21 and 5.182 eV for **M1**; 1.57, −4.34 and 5.998 eV for **M2**; 1.51, −4.36 and 6.295 eV for **M3**; 1.72, −4.15, and 5.009 eV for **M4**;1.69, −4.12 and 5.00 eV for **M5**; and 1.58, −4.48 and 6.351 eV for **M6**. The μ and ω values for **M6** further indicated that the compound would be a good electron acceptor with strong electron pulling effects toward the two NO_2_ groups. This finding was in agreement with the effect of the NO_2_ group observed on 4-(3-(2-amino-3,5-dibromophenyl)-1-(4-nitrobenzoyl)-4,5-dihydro-1*H*-pyrazol-5-yl)benzonitrile by Santhi and Bharathi.^28^ The electron donating power (ω^−^) and electron accepting power (ω^+^) describe the tendency of a molecule to release electrons and to accept electrons, respectively; a smaller ω^−^ indicates a better donor of electron density, and a greater ω^+^ indicates better accepting electron capacity. The values for ω^−^ demonstrated that **M1, M4** and **M5** were good electron donors, whereas ω^+^ indicated the tendency of **M2, M3** and **M5** to be good electron acceptors, in line with the μ and ω values (Table 4).

**Table 4:** Molecular orbitals and reactivity indices.

Parameter	**M1**	**M2**	**M3**	**M4**	**M5**	**M6**	**Ma**
HOMO (eV)	−5.92	−5.91	−5.87	−5.86	−5.79	−6.06	−6.10
LUMO (eV)	−2.50	−2.77	−2.85	−2.43	−2.42	−2.90	−2.26
HOMO−1 (eV)	−6.71	−6.62	−6.10	−6.41	−6.32	−6.90	−6.79
HOMO−2 (eV)	−7.02	−6.91	−6,32	−6.61	−6.51	−7.60	−7.21
LUMO+1 (eV)	−2.01	−2.02	2.41	−2.03	−1.92	−2.61	−1.78
ΔE_g_ (eV)	3.42	3.14	3.02	3.43	3.37	3.16	3.83
η (eV)	1.71	1.57	1.51	1.715	1.685	1.58	1.92
μ (eV)	−4.21	−4.34	−4.36	−4.145	−4.105	−4.48	−4.18
χ (eV)	4.21	4.34	4.36	4.145	4.105	4.48	4.18
ω (eV)	5.182	5.998	6.295	5.009	5.00	6.351	4.562
ω+ (eV)	3.291	4.025	4.303	3.151	3.158	4.309	2.700
ω− (eV)	7.501	8.365	8.663	7.296	7.263	8.789	6.880
σ (eV^−1^)	0.855	0.785	0.755	0.8575	0.8425	0.7900	0.5208
ΔE_n_ (eV)	1.827	2.444	2.670	1.722	1.7378	2.661	1.334
ΔE_e_ (eV)	10.247	11.124	11.410	10.011	9.948	11.621	9.715

Ma = Theoretical data of the compound from Ref.^28^.

The absorption peaks, oscillator strength and percentage of the molecular orbitals involved in transitions, calculated for **M1–M6** at B3LYP/6-31G (p,d), are displayed in Table 5. The transition probability, as measured by oscillator strength (OS), corresponded to the fraction of negative charges (electrons) that accomplished a given transition. OS values <0.005 were considered to indicate transitions emanating from low absorption bands in the studied compounds; thus, only transitions with OS > 0.005 were considered in this study. **M1** showed four strong absorptions, at 304.69, 333.69, 349.07 and 408.24 nm, arising from HOMO-2 → LUMO (94%), HOMO-1 → LUMO (96%), HOMO → LUMO + 1 (89%) and HOMO → LUMO (98%), respectively; the longest *λ*_max_ was characterized as a π–π∗ transition arising from HOMO → LUMO. For **M2**, five strong absorption peaks were identified, at 305.98, 337.57, 341.62, 356.73 and 453.95 nm, with an OS of 0.0463, 0.0081, 0.2840, 0.0359 and 0.0285, respectively. The molecular orbitals’ percentage contributions to these transitions were as follows: HOMO-3 → LUMO (42%) and HOMO-4 → LUMO (35%) for 305.98 nm; HOMO-2 → LUMO (87%) for 337.57 nm; HOMO → LUMO+1 86% for 342 nm; HOMO-2 → LUMO 99% for 358 nm; and HOMO → LUMO 99% for 453.95 nm.

**Table 5:** Calculated absorption peaks, oscillation strength and molecular orbitals involved in transitions for **M1–M6**.

*λ*_max_ (nm)	Oscillation strength	MO involved in transitions
**M1**		
304.60	0.0349	HOMO-2 → LUMO 94%
333.69	0.0125	HOMO-1 → LUMO 96%
349.07	0.2918	HOMO → LUMO+1 89%
408.24	0.0105	HOMO → LUMO 98% triplet
**M2**		
305.98	0.0463	HOMO-3 → LUMO 42% HOMO-4 → LUMO 35%
337.57	0.0081	HOMO-2 → LUMO 87%
341.62	0.2840	HOMO → LUMO+1 86%
356.73	0.0359	HOMO-1 → LUMO 99%
453.97	0.0285	HOMO → LUMO 99% triplet
**M3**		
408.96	0.0131	HOMO-2 → LUMO 81%
420.81	0.0177	HOMO-5 → LUMO 67%
426.26	0.0243	HOMO → LUMO+1 44% HOMO-1 → LUMO+1 33%
439.54	0.0394	HOMO-1 → LUMO+1 45% HOMO → LUMO+1 38%
445.90	0.0082	HOMO-1 → LUMO 88%
494.49	0.0059	HOMO → LUMO 96% triplet
**M4**		
309.69	0.0983	HOMO-1 → LUMO+1 94%
332.48	0.0142	HOMO-2 → LUMO 92%
341.18	0.0057	HOMO-1 → LUMO 95%
347.16	0.3138	HOMO → LUMO+1 86%
409.31	0.0109	HOMO → LUMO 98% triplet
**M5**		
313.41	0.0901	HOMO-1 → LUMO+1 96%
337.82	0.0253	HOMO-2 → LUMO 92%
347.01	0.2753	HOMO → LUMO+1 79%
351.40	0.0416	HOMO-1 → LUMO 93%
417.91	0.0115	HOMO → LUMO 98% triplet
**M6**		
329.32	0.0199	HOMO-1 → LUMO+1 97%
342.96	0.2651	HOMO → LUMO+2 89%
349.68	0.0460	HOMO-1 → LUMO 99%
407.32	0.0125	HOMO → LUMO+1 99% triplet
451.04	0.0267	HOMO → LUMO 99% triplet

In addition, **M3** showed five strong absorption peaks, with HOMO-2 → LUMO (81%) for 408.96 nm; HOMO-5 → LUMO (67%) for 420.81 nm; HOMO-2 → LUMO+1 (44%) and HOMO-1 → LUMO +1 (33%) for 426.26 nm; HOMO-1 → LUMO+1 (45%) and HOMO → LUMO+1 (38%) for 439.54 nm; HOMO-1 → LUMO (88%) for 445.90 nm; and HOMO → LUMO (96%) for 494.49 nm, arising from a low absorption band of 0.0059 OS. For **M4**, 309.69, 332.48, 347.16 and 409.31 nm were the four absorption peaks with OS higher than 0.005, arising from HOMO-1 → LUMO+1 (94%), HOMO-2 → LUMO (92%), HOMO → LUMO+1 (86%) and HOMO → LUMO (98%), respectively. Likewise, 313.41, 337.82, 347.01, 351.47 and 417.91 nm were five absorption peaks with OS higher than 0.005 arising from HOMO-1 → LUMO+1 (96%), HOMO-2 → LUMO (92%), HOMO → LUMO+1 (79%), HOMO-1 → LUMO (93%) and HOMO → LUMO (98%), respectively, for **M5**. The 417.91 nm absorption peak with the highest OS value was characterized as a π–π∗ transition. For **M6**, five strong absorption peaks at 329.32, 342.96, 349.68, 407.32 and 451.04 nm were identified with OS values of 0.0199, 0.2651, 0.0460, 0.0125 and 0.0267, respectively: HOMO-1 → LUMO+1 (97%) for 329 nm; HOMO → LUMO+2 (89%) for 324.9 nm; HOMO-1 → LUMO (99%) for 349.68 nm; HOMO → LUMO+1 (99%) for 407 nm; and HOMO → LUMO (99%) for 451 nm. The absorption peak with the highest OS was characterized as a π–π∗ transition, and the absorption peak next to the highest OS was characterized as an n–π∗ transition. All the compounds had one or two triplet transitions, thus potentially indicating that they possessed both orbital unpaired and spin unpaired electrons. Consequently, singlet transitions with sufficiently long lifetimes might have led to de-inversion of the spin of some of the electrons, thus generating a triplet.

### Molecular electrostatic potential analysis

**Figure 2:**
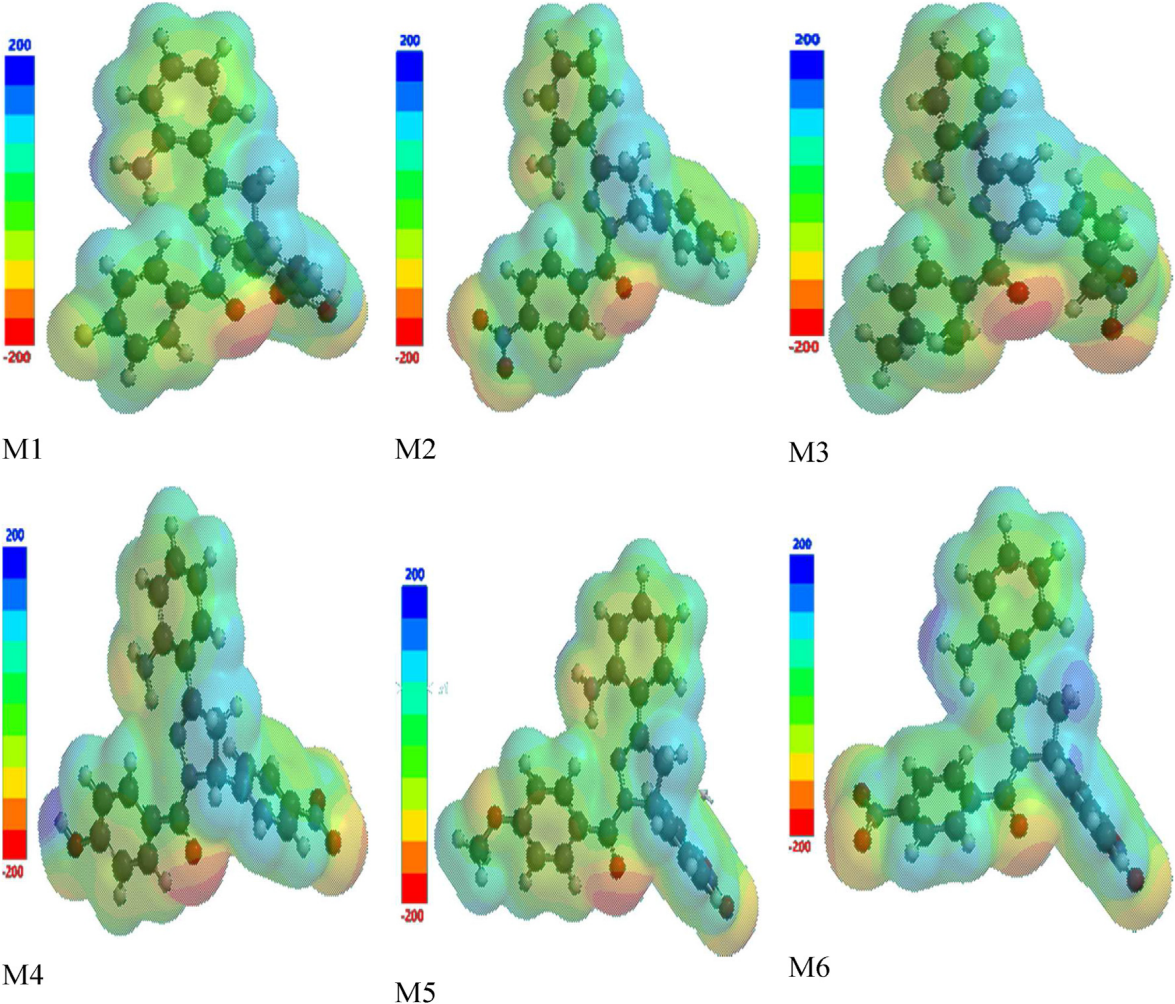
Molecular electrostatic potential (MEP) diagram of compounds **M1**–**M6**.

The static distribution of charge density is associated with the electrostatic potential map (MEP) distribution of charge on a molecule, and is a useful parameter for analyzing and predicting the responsiveness of a molecule toward an incoming electrophile or nucleophile during reaction initiation.^47^ This parameter has been successfully used to explain stacking and self-assembly of polymeric molecules and dyes, and the orientation of molecules in three-dimensional crystals.^48^ The MEP was simulated according to the optimized geometry obtained from DFT calculation to predict electrophilic and nucleophilic sites of attack. In Figure 2, blue indicates a positive region indicating electrophilic centers/electron-deficient areas; red represents the negative regions (areas with excess electrons) for nucleophilic reactivity/electron-rich centers of a molecule; and green represents regions with essentially zero potential.^49^, ^50^, ^51^ Generally, the MEP ranged from red to orange to yellow to green to blue (Figure 1). The negative (red) regions were located at the carbonyl oxygen and cyano group, thus indicating the most probable sites for electrophilic attack. The regions with excess electrons could result in intermolecular pulling of positive regions of nearby molecules, thus distorting the π–π stacking arrangement of these molecules.

### Vibration frequencies

Vibration frequency is a powerful and effective technique for identification of the organic functional groups of organic compounds; it can distinguish a molecular conformer form either its tautomer or isomer. Comparison of experimental and calculated vibrational modes with appropriate functional group assignment provides meaningful and useful information for understanding fairly complex systems.^34^ In addition, when experimental data are not available, the theoretical calculated vibration frequencies can be used with a reasonable level of accuracy to understand the effects of functional groups on molecules. Scaling of the calculated wave numbers increases the reliability and utility of the calculated frequency^28^^,^^52^; thus, a 0.9608 scaling factor was used in this work. The DFT-B3LYP/6-31G(d,p) level of calculation was used to determine the vibration frequencies of **M1–M6** compounds, and the results were compared with those of 4-(3-(2-amino-3,5-dibromophenyl)-1-(benzoyl)-4,5-dihydro-1*H*-pyrazol-5-yl)benzonitriles.^28^ The scaled and unscaled vibration frequencies calculated with the DFT method are shown in Table 6. The calculated symmetric and asymmetric N–H stretching vibrations were 3394 and 3551 cm^−1^ for **M1**; 3399 and 3552 cm^−1^ for **M2**; 3385 and 3542 cm^−1^ for **M3**; 3389 and 3549 cm^−1^ for **M4**; 3391 and 3551 cm^−1^ for **M5**; and 3399 and 3551 cm^−1^ for **M6**. The value has been experimentally observed to be 3433 cm^−1^ and calculated to be 3452 cm^−1^ for **Ma**.^28^ Experimental observations have been conducted at 3390, 3378 and 3360 cm^−1^ in 1-((1,3-diphenyl-1*H*-pyrazol-4-yl)methylene)-4-phenylsemicarbazide, 4-phenyl-1-((1-phenyl-3-p-tolyl-1*H*-pyrazol-4-yl)methylene)semicarbazide and 1-((3-(4-hydroxyphenyl)-1-phenyl-1*H*-pyrazol-4-yl)methylene)-4-phenylsemicarbazide, respectively.^8^ The impure in plane bending N–H vibration (βN-H) was blended with vC = C stretching, as shown in Table 6; thus, the βN-H was calculated to be 1345, 1394, 1349, 1344, 1346 and 1345 cm^−1^ for **M1–M6**, respectively, but has been reported to be 1398 and 1402 cm^−1^ for **Ma**.^28^

**Table 6:** Calculated IR frequencies (cm^−1^) at B3LYP/6-31G(d,p).

Assignment	**M1**		**M2**		**M3**		**M4**		**M5**		**M6**		**Ma**	
	Unscaled	Scaled	Unscaled	Scaled	Unscaled	Scaled	Unscaled	Scaled	Unscaled	Scaled	Unscaled	Scaled	Expt	Scaled
νN-H_syn_	3532	3394	3538	3399	3523	3385	3527	3389	3529	3391	3538	3399	3433	3452
νN-H_Asyn_	3696	3551	3697	3552	3687	3542	3694	3549	3696	3551	3696	3551	–	
νC-H_syn_	3110	2988	3102	2980	3099	2978	3106	2984	3113	2991	3106	2984	2924	2980
νC-H_Asyn_	3239	3112	3250	3123	3195	3070	3234	3107	3233	3106	3231	3104	3071	3104
νC = O	1723	1655	1730	1662	1686	1620	1720	1653	1725	1657	1731	1663	1655	1646
νC = N	1642	1578	1637	1573	1632	1568	1634	1570	1634	1570	1639	1575	1619	1622
νC = C, βN-H	1345	1292	1347	1294	1349	1296	1344	1291	1346	1293	1345	1292	1398	1402
vC = C	1618	1555	1614	1551	1579	1517	1617	1554	1618	1555	1616	1553	1329	1353
βC-H	1192, 1177, 1172	1145, 1131, 1127	1213, 1188, 1176	1165, 1142, 1130	1197, 1189, 1178	1150, 1143, 1132	1203, 1188, 1176	1156, 1141, 1130	1201, 1183, 1171	1154, 1137, 1125	1203, 1183, 1177	1155, 1137, 1131	1165	1175
τC-H	998 920, 886	959, 884, 851	991, 948, 843	952, 910, 809	996, 952 856,	957, 915, 822	966, 956, 886	928, 918, 790	979, 964, 895	941, 926, 860	998, 964, 890	959, 926, 855	987	980
νC-F	1287	1237	1287	1237	–	–	–	–	–	–	–	–	1223	–
νC-NO_2_	1397	1342	1397	1342	1392	1337	1397	1342	1397	1342	1397	1342	1450a, 1394a	1489a, 1397a
νNO_2_	1671	1605	1668	1603	1631	1567	1671	1605	1670	1605	1669, 1619, 1616	1604, 1556, 1553		
vNH_2_	1624	1560	1623	1559	1624	1560	1623	1559	1820	1556	1623	1559		
vC-NH_2_	1658	1593	1662	1597	1654	1589	1664	1599	1663	1598	1661	1596		
νOH							3819	3669						

ν = stretching; β = in-plane bending: τ = out-of-plane bending, Sci = scissors; Ma and a = 4-(3-(2-amino-3,5-dibromophenyl)-1-(4-nitrobenzoyl)-4,5-dihydro-1*H*-pyrazol-5-yl)benzonitrile.^28^.

The aromatic C–H stretching vibrations for the compound were in the range of 3250–3102 cm^−1^, but were scaled to be in the region 2984–2978 cm^−1^. The C–H asymmetric stretching vibration for compounds **M1–M6** was calculated to be 3112, 3195, 3070, 3107, 3106 and 3104 cm^−1^; the asymmetric C–H symmetric vibrations were 2988, 2980, 2978, 2984, 2991 and 2984 cm^−1^ for **M1–M6**, respectively. The C–H in-plane bending (βC-H) vibrations were 1145, 1131 and 1127 cm^−1^ for **M1**; 1165, 1142 and 1130 cm^−1^ for **M2**; 1150, 1143 and 1132 cm^−1^ for **M3**; 1156, 1141 and 1130 cm^−1^ for **M4**; 1154, 1137 and 1125 cm^−1^ for **M5**; and 1155, 1137 and 1131 cm^−1^ for **M6**. The βC-H vibration for 4-(3-(2-amino-3,5-dibromophenyl)-1-(benzoyl)-4,5-dihydro-1*H*-pyrazol-5-yl)benzonitriles has been experimentally observed at 1165 cm^−1^ and calculated to be 1175 cm^−1^.^28^ The C–H out-of-plane (τC-H) vibrations appeared in the region of 959–851 cm^−1^ for **M1**; 952–809 cm^−1^ for **M2**; 957–822 cm^−1^ for **M3**; 928–790 cm^−1^ for **M4**; 941–860 cm^−1^ for **M5**; and 959–855 cm^−1^ for **M6**.

The C = O stretching vibrations appeared in the region of 1655 cm^−1^ for **M1**; 1662 cm^−1^ for **M2**; 1620 cm^−1^ for **M3**; 1653 cm^−1^ for **M4**; 1657 cm^−1^ for **M5**; and 1663 cm^−1^ for **M6**. These vibrations have been observed at 1662 cm^−1^ for *N*-[(4-chlorophenyl)]-4-oxo-4-[oxy] butane amide,^53^ 1637 cm^−1^ for diethyl 1*H*-pyrazole-3,5-dicarboxylate,^25^ 1660 cm^−1^,^28^ 1701 cm^−1^ for 4-chloro-3,5-dimethyl-1*H*-pyrazol-1-yl) (p-tolyl) methanone^26^ and 1683 cm^−1^ for 4-phenyl-1-((1-phenyl-3-p-tolyl-1*H*-pyrazol-4-yl)methylene)semicarbazide.^8^ The calculated vC-NO_2_ stretching was 1337 cm^−1^ for **M3** but was 1342 cm^−1^ for other compounds. The νNO_2_ (acceptor group) stretching was calculated to be 1605, 1603, 1567, 1605 and 1605 cm^−1^ for **M1–M5**, respectively; meanwhile, M6 showed three vibrational modes for νNO_2_, at 1604, 1556 and 1553 cm^−1^. However, these bands were calculated for *N*-methyl-*N*-(2,4,6-trinitrophenyl) nitramide to be in the region of 1633–1591 cm^−1^ and 1388–1348 cm^−1^, and were assigned to vNO_2_ asymmetric and symmetric stretching vibrations, respectively.^54^ The vNH_2_ group (electron donor) stretching was 1560 cm^−1^ for **M1** and **M3**; 1559 cm^−1^ for **M2, M4** and **M6**; and 1556 cm^−1^ for **M5**. Generally, aromatic compounds containing fluorine show vC–F stretching in the region 1000–1400 cm^−1^.^55^ However, compounds **M1** and **M2** showed these vibrations at 1237 cm^−1^ and have been reported at 1223 cm^−1^.^28^

### Polarizability and hyperpolarizability

The static polarizability (α), hyperpolarizability (β) and electric dipole moment (μ) based on the finite field method were calculated for the six compounds at DFT B3LYP/6-31G(d,p). The total static dipole moment (μ), mean polarizability (α_o_) and mean first hyperpolarizability (β_0_) were defined by using the x,y,z component, as shown in equations 8, 9, 10, 11, 12, 13, 14):

8 μTotal=(μx2+μy2+μz2)12

9 $$\alpha_{0}=1/3\left(\alpha_{xx}+\alpha_{yy}+\alpha_{zz}\right)$$

10 $$\alpha^{2}=1/2\left[{\left(\alpha_{xx}-\alpha_{yy}\right)}^{2}+{\left(\alpha_{zz}-\alpha_{xx}\right)}^{2}+6\left(\alpha_{xx}^{2}+\alpha_{yy}^{2}+\alpha_{zz}^{2}\right)\right]$$

11 β0=βTotal=(βx2+βy2+βz2)12

12 $$\beta_{x}^{2}={\left(\beta xxx+\beta xyy+\beta xzz\right)}^{2}$$

13 $$\beta_{y}^{2}={\left(\beta yyy+\beta yzz+\beta yxx\right)}^{2}$$

14 $$\beta_{z}^{2}={\left(\beta zzz+\beta zxx+\beta zyy\right)}^{2}$$

The presence of electron donating and electron withdrawing groups on π-conjugated molecules changes the ground state charge distribution and enhances asymmetric polarization of the molecules. Consequently, large nonlinear responses are correlated with a rapid response time; therefore, these molecules are desirable candidates for NLO applications.^56^^,^^57^ Any molecule with a minimum value of 4.187944 × 1^−30^ esu for the first hyperpolarizability is considered a good candidate for NLO applications.^58^ Therefore, these molecules’ NLO properties, polarizability and hyperpolarizability were assessed. The dipole moment, an essential parameter explaining the intermolecular interactions of molecules, is expected to have a higher value with stronger intermolecular interactions. The dipole moment values calculated for **M1–M6** were 4.57, 4.06, 5.47, 6.45, 5.32 and 5.14 D (1D = 3.34 × 10^−34^ C m), respectively. The α_o_ calculated for **M1–M6** was 3.29, 3.28, 3.26, 3.26, 3.28 and 3.63 × 10^−23^ esu, respectively. The α_o_ for Ma (4-[3-(2-amino-3,5-dibromophenyl)-1-(4-fluorobenzoyl)-4,5-dihydro-1*H*-pyrazol-5-yl]benzonitrile) has been reported to be 3.31 × 10^−23^ esu.^28^ However, the β_0_ for **M1–M6** was 6.04, 5.21, 6.15, 6.74, 6.02 and 7.26 3.31 × 10^−30^ esu, respectively, and has been reported to be 8.47 × 10^−30^ esu for **Ma**.^28^ The β_0_ values for the studied compounds were lower than that of Ma, but approximately 16 times higher than that of urea (0.372 × 10^−30^ esu).^57^ The model compounds had higher β_0_ values than those of diphenylmethylidene-5-methyl-1*H*-pyrazole-3-carbohydrazide (β_0_ = 2.08 × 10^−30^ esu)^59^ and diethyl-1-*H*-pyrazole-3,5-dicarboxylate (β_0_ = 1.01 × 10^−30^ esu).^24^ Interchanging the position of NO_2_ and F, as shown in **M1** and **M2**, led to a decrease in β_0_ by 0.83 × 10^−30^ esu and an increase in absorption wavelength (*λ*_max_) by 75 nm in **M2**. Thus, the position, nature and point of attachment of substituents on the model compound strongly affect the properties of the compound.^57^ In addition, **M6** with two NO_2_ groups increased β_0_; therefore, the presence of an electron withdrawing group (nitro) on the phenyl ring contributed to higher hyperpolarizability values, possibly because of an inductive effect of the electron withdrawing group on the electronic density in the molecule.^28^ Our results showed that **M1–M6** compounds may be suitable for NLO applications, and the magnitude of molecular hyperpolarizability was improved by functional group modification (Table 7).

**Table 7:** Dipole moment, polarizability and hyperpolarizability of **M1–M6.**

Dipole moment						
Parameter	**M1**	**M2**	**M3**	**M4**	**M5**	**M6**
μ_x_	3.2717	2.4264	−4.4201	4.22	1.2246	−3.7596
μ_y_	−2.0652	−2.9515	−2.3989	−4.2089	4.9235	1.4025
μ_z_	−2.4399	1.3697	2.1446	−2.4754	1.5946	3.2168
μ_tot_	4.57	4.06	5.47	6.45	5.32	5.14
Polarizability/a.u.						
α_xx_	−242.284	−236.429	−258.740	−246.939	−237.601	−255.538
α_xy_	11.3659	14.9274	−13.1037	6.41700	−31.0350	−6.7400
α_yy_	−218.433	−225.704	−184.970	−203.456	−211.066	−262.856
α_xz_	12.0644	−4.6114	10.8756	12.2329	−4.94560	13.6039
α_yz_	−16.3197	−2.0474	10.8167	−16.7704	−25.3816	6.80760
α_zz_	−206.567	−202.342	−216.967	−208.855	−215.010	−217.261
α_tot_	−222.428	−221.491	−220.225	−219.749	−221.226	−245.218
α × 10^−23^ esu	3.29	3.28	3.26	3.26	3.28	3.63
Hyperpolarizability/a.u.						
β_xxx_	415.4398	307.9092	−499.813	399.1294	296.4138	−451.768
β_xxy_	−143.263	−61.1607	−94.8057	−99.1944	215.4269	−151.772
β_xyy_	160.3521	223.159	−36.6282	215.598	85.0875	−278.063
β_yyy_	−5.6029	−50.0772	−72.1537	−181.966	262.2293	244.9397
β_xxz_	−37.1159	8.9984	42.1252	−29.5710	−7.1656	55.6498
β_xyz_	84.942	−9.2125	34.8642	81.0249	105.6754	53.0815
β_yyz_	−31.9896	53.7171	−11.3035	−51.069	27.7063	100.8334
β_xzz_	103.2154	62.224	−149.912	109.4585	108.3569	−91.1906
β_yzz_	−4.1993	13.766	−18.9092	2.0788	18.0156	−6.0768
βzzz	5.8459	−20.1728	−5.7925	4.1569	−6.0768	−0.3585
β_tot_	698.9145	602.749	711.516	779.86	697.037	840.259
β × 10^−30^ esu	6.04	5.21	6.15	6.74	6.02	7.26

For (α): 1 a.u. = 0.1482 × 10^−24^ esu and for (β): 1 a.u. = 8.6393 × 10^−33^ esu).

### Molecular docking

The antihypertensive and antioxidant properties of the studied pyrazole derivatives (**M1–M6**) were investigated via molecular docking, and the obtained results were compared with the results for rolipram and taurine. The antihypertensive activity of the compounds was evaluated by considering their inhibitory activity against phosphodiesterases (PDEs). PDEs are enzymes participating in cAMP and cGMP homeostasis by acting on phosphodiester bonds.^60^^,^^61^ When PDE is inhibited, the cAMP and cGMP levels increase, thereby decreasing calcium levels in cells. Consequently, blood vessels are vasodilated with relaxing of the blood vessels, and the risk of hypertension markedly decreases.^62^ However, phosphodiesterase 4 (**PDE4**) has been reported to be the main enzyme in the hydrolysis of cAMP, another mediator that controls pro-inflammation and anti-inflammation.^61^^,^^62^ To investigate the PDE inhibitory activity of the compounds, we performed molecular docking of the compounds on PDE4 (**PDB ID:** **1RO6**) downloaded from the Protein Data Bank. The downloaded **1RO6** was complexed with the rolipram drug, a selective PDE4 inhibitor that increases the quantity of cAMP in immune and nerve cells^63^^,^^64^ and compared the docking results.

Antioxidants are common food additives that inhibit cellular damage mainly through their free radical scavenging ability.^64^^,^^65^ Free radicals are reactive oxygen species produced in the body through various metabolic processes, in phagocytosis, in prostaglandin synthesis and in the cytochrome P-450 system, as a result of exposure to different physiochemical conditions or pathological states.^66^ Excessive free radicals in the body lead to a condition known as oxidative stress, which harmfully alters proteins, lipids and DNA, and can initiate the progression of pathologies including immune system deterioration, atherosclerosis and abnormal cell growth leading to nucleofugality cancer.^66^ Analysis of pyrazoline derivatives has indicated that they are promising antioxidants.^67^, ^68^, ^69^ Therefore, we examined the model compounds for their antioxidant ability by docking them against a dehydrogenase inhibitor downloaded from the Protein Data Bank (**PDB ID:** **5ADH****)**. Taurine is an antioxidant involved in protection of hepatic tissue by deactivating reactive oxygen species, thereby removing formation of osmoregulation, calcium homeostasis, lipid peroxidation and protein carbonyl formation, detoxification, cytoprotection and neuromodulation.^70^, ^71^, ^72^ Reports have indicated that taurine concentrations are inversely associated with diabetes complications.^73^, ^74^, ^75^ The optimized structures of the model compounds were used for docking simulations. The docking was performed with AutoDock Tool 1.5.6 and AutoDock Vina 1.1.2; proteins were treated; and molecular interactions between receptors and ligands were visualized with Edupymol version 1.7.4.4 and BIOVIA Discovery studio 2019, as previously reported.^76^, ^77^, ^78^, ^79^, ^80^, ^81^, ^82^, ^83^, ^84^ The grid box using AutoDock tool before use of AutoDock Vina for docking calculations was as follows: center (X = 4.834, Y = 15.305, Z = 24.227) and size (X = 64, Y = 52, Z = 74) for **5ADH**, and (X = 32.382, Y = 72.334, Z = 31.711) and size (X = 62, Y = 58, Z = 66) for **1RO6**, with default exhaustiveness (exhaustiveness = 8) for steady docking calculation speed.

A recent study has indicated that molecular docking of pyrazole derivatives such as carboxy pyrazole derivatives with various cancer cells (breast, MCF-7; bone marrow, K-562; and cervix, HeLa),^6^ aryl pyrazoles with tyrosinase enzyme,^85^ pyrazole-phenyl semicarbazone derivatives with α-glucosidase,^8^ imidazole–pyrazole conjugates with α-glucosidase^10^ and 4-aryl-N-(5-methyl-1*H*-pyrazol-3-yl)benzamides with *Acinetobacter* *baumannii* protein^11^ was in agreement with experimental observations, and has also detailed the nature of the protein-ligand interactions. The use of molecular docking for in silico screening of bioactivity of heterorganic compounds, as well as drug design and discovery, has become frequent and relevant in pharmacology. Therefore, docking serves as a reliable and time saving method for simulation of binding poses of ligand conformations in the active sites of receptors, and calculation of the binding affinity and interactions of protein-ligand complexes.^86^

**Figure 3:**
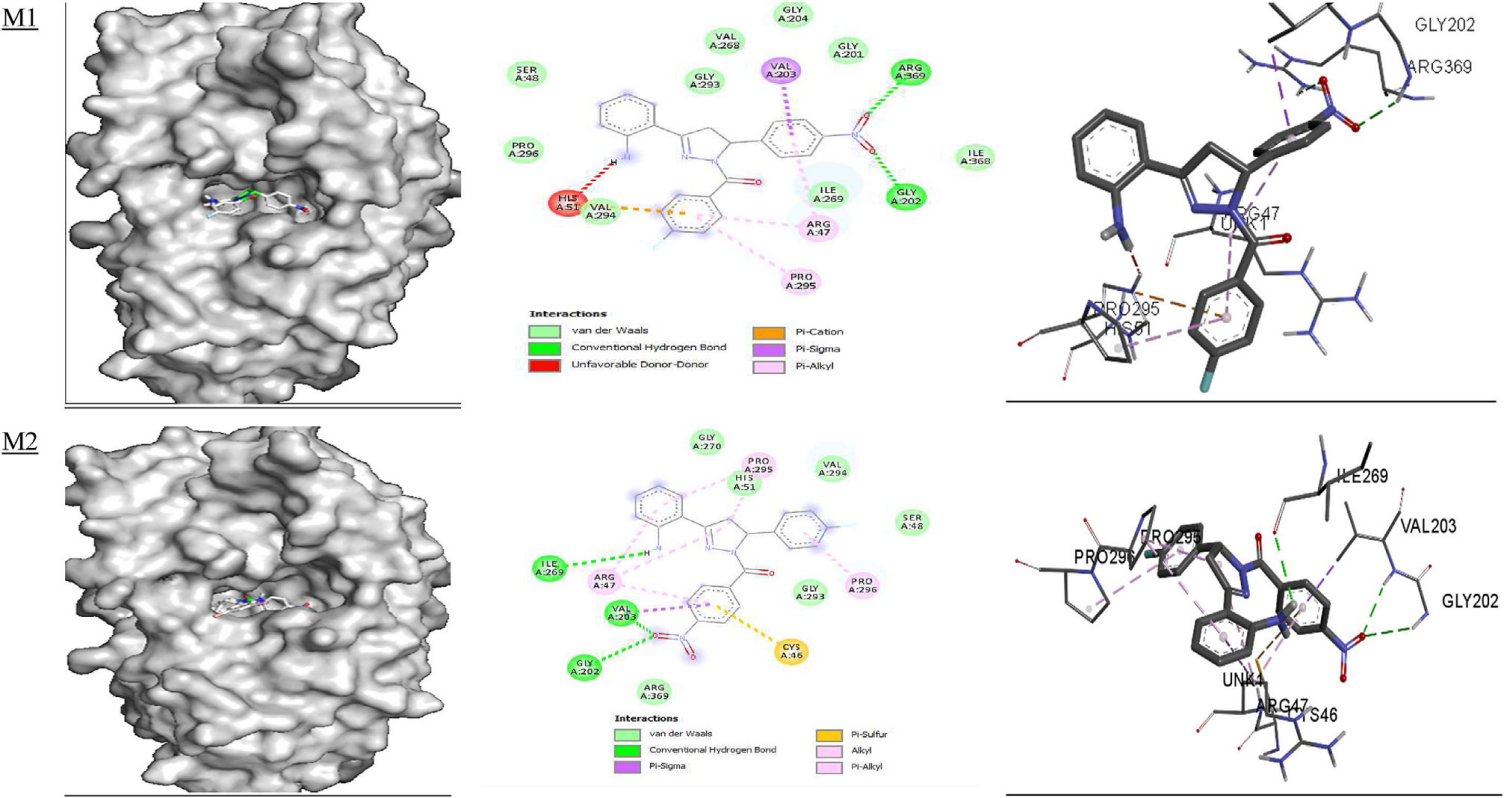

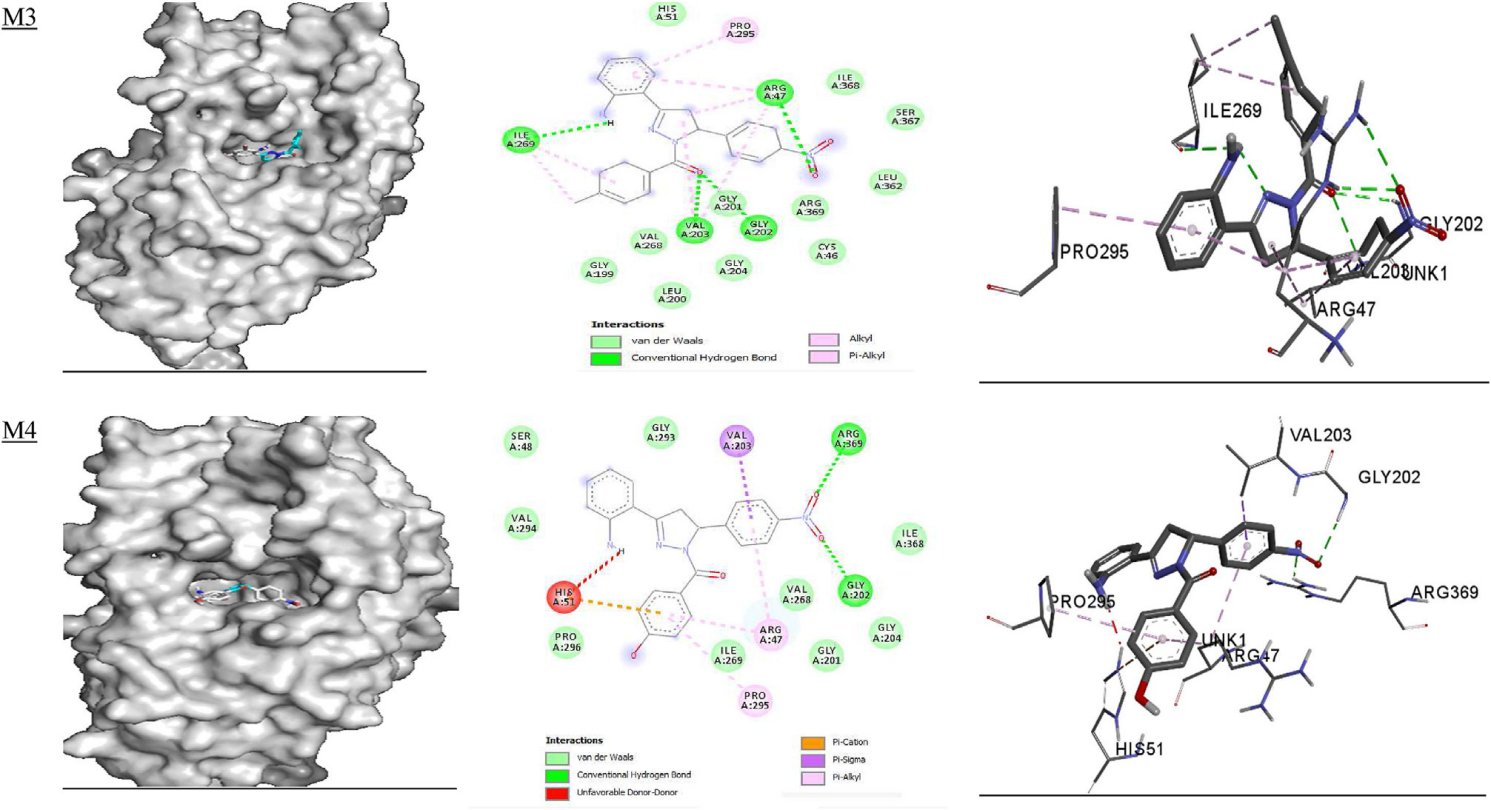

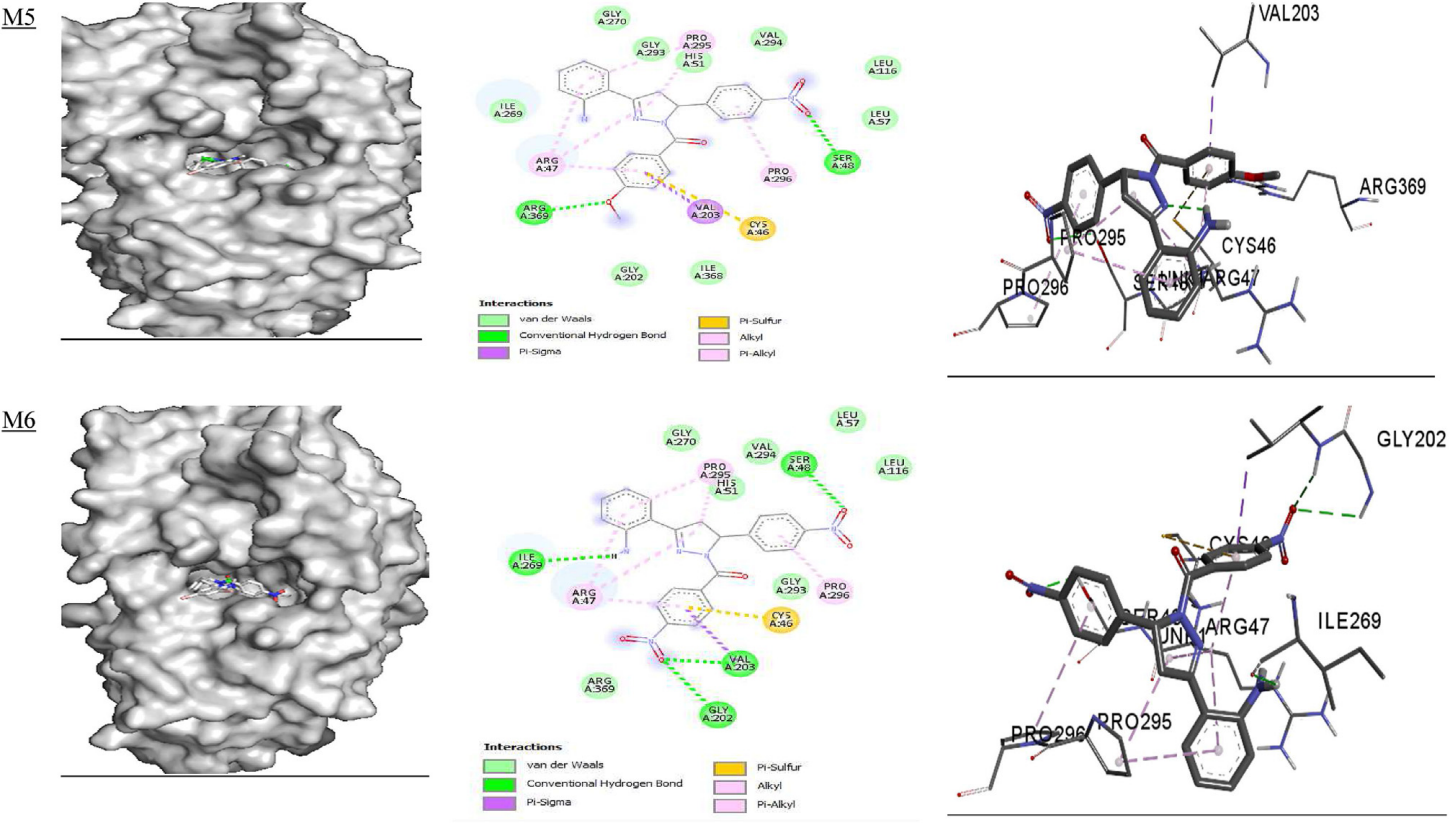

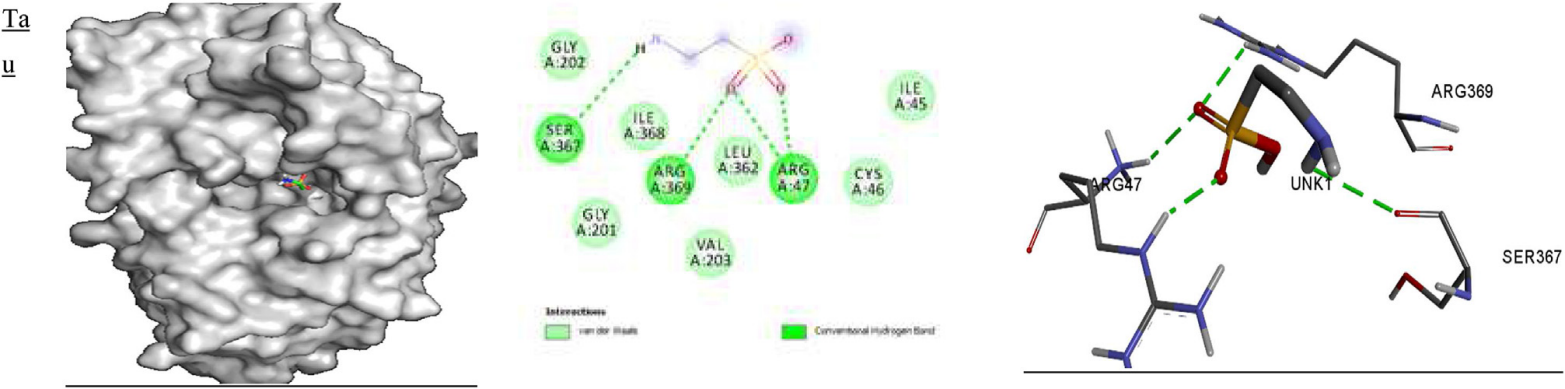
Docked complexes of the **5ADH** receptor with compounds **M1–M6**, showing interactions in the binding pocket.

The binding affinity of the stable ligands docked with dehydrogenase inhibitor (**PDB ID:** **5ADH****)** ranged from −8.8 to 9.3 kcal/mol: **M1** (−9.0 kcal/mol), **M2** and **M3** (−9.3 kcal/mol) and **M4, M5** and **M6** (−8.8 kcal/mol). Similar binding affinities (−8.3 to −9.5 kcal/mol) have been reported for 1-benzyl-2-phenyl-1*H*-benzimidazole derivatives docked with dehydrogenase (**PDB ID:** **5ADH****)**.^87^ The binding affinity calculated for taurine was −3.7 kcal/mol, as shown in Table 8, thus indicating that these compounds may be excellent inhibitors for APO-liver dehydrogenase and thus possess good antioxidant properties. Ligand interactions with the binding pocket of dehydrogenase (**PDB ID:** **5ADH**) revealed that ARG 369 and ARG 202 are involved in hydrogen bond interactions with the NO_2_ group of **M1;** ARG 295 and ARG 47 are involved in a π-alkyl interaction; Val 294 is involved in a π-cation interaction, and VAL 203 is involved in π-sigma interactions with **M1.** The amino acid residues GLY 202, VAL 203 and ILE 269 are involved in hydrogen bond interactions; PRO 295, ARG 47 and PRO 296 are involved in π-alkyl interactions; VAL 203 is involved in π-sigma interactions; and CYS 46 is involved in π-sulfur interactions with **M2.** In addition, GLY 202, VAL 203, ARG 47 and ILE 269 are involved in hydrogen bonding, and ILE 269, VAL 203, GLY 202, PRO 295 and ARG 47 form π-alkyl interactions with **M3**. PRO 295 and ARG 47 are involved in π-alkyl interactions; VAL 203 is involved in π-sigma interactions; ARG 369 and GLY 202 form hydrogen bond interactions, and HIS 51 is involved in π-cation interactions with **M4**. Amino acid residues ARG 47, PRO 295 and PRO 296 are involved in π-alkyl interactions; SER 48 and ARG 369 are involved in hydrogen bonding; VAL 203 forms a π-sigma interaction; and CYS 46 forms π-sulfur interactions with **M5.** Furthermore, SER 48, VAL 203 and ILE 269 are involved in hydrogen bonding; ARG 47, PRO 295 and PRO 296 form π-alkyl interactions; and VAL 203 is involved in a **π**-sigma interaction with **M6** (Table 8 and Figure 3). Taurine is involved in hydrogen bonding with SER 367, ARG 369 and ARG 47, and also participates in Van der Waals interactions with VAL 203 and CYS 46**.** Similar binding modes for protein-ligand interactions have been observed for 1-benzyl-2-phenyl-1*H*-benzimidazole derivatives docked with dehydrogenase (**PDB ID:** **5ADH****)**, and ILE 269, VAL 203, GLY 202, PRO 295 and ARG 47 amino acid residues have been found to be involved in the interactions.^87^

**Table 8:** Binding affinity (**ΔG**) and hydrogen bonding interactions of the 5ADH and 1RO6 receptors with compounds **M1–M6**.

Ligand	5ADH receptor				1RO6 receptor				
	Binding affinity ΔG (kcal/mol)	Inhibition constant Ki (μM)	H-bond with ligands	H-bond distance (Å)	Binding affinity ΔG (kcal/mol)		Inhibition constant Ki (μM)	H-bond with ligands	H-bond distance (Å)
**M1**	−9.0	0.25	ILE'269 VAL'294 GLY'202 ARG'269 ILE'368	2.9 2.4 2.3 2.1 3.3	−9.7		0.07	GLU'304 ASP'392 ASP'275	3.2 3.2 3.5
**M2**	−9.3	0.15	ILE'269 VAL'203	2.7 2.5	−9.2		0.17	SER'282 GLN'417	2.7 2.4
**M3**	−9.3	0.15	ILE'269 ARG'47 SER'367 ILE'368 GLY'202	2.7 2.4, 2.6 3.2 3.2 2.7	−9.4		0.12	HIS'234 ASN'395 GLN'443	2.2 2.0 3.3, 2.0
**M4**	−8.8	0.35	ILE'269 VAL'294 ARG'369 ILE'368 GLY'202	3.0 2.8 2.1 2.3 2.3	−9.9		0.05	GLU'304 GLN'417 GLY'280 ASP'392 ASP'275	3.5 2.8 2.8 3.1 3.5
**M5**	−8.8	0.35	ARG'369	2.2	−8.5		0.58	GLU'304 ASP'275 HIS'234	2.4 3.5 2.5
**M6**	−8.8	0.35	SER'48 ILE'269 VAL'203	2.6 2.6 2.5	−8.9		0.29	THR'345 HIS'307 HIS'234 ASN'395	3.3 2.5 2.4 2.4
**Taurine**	−3.7		SER 367 ARG 369 ARG 47	2.6 2.4 2.3	**Rolipram**	−8.8	0.35	ASP'392 HIS'234 GLN'443	3.6 2.1 3.3, 2.4, 3.3

**Figure 4:**
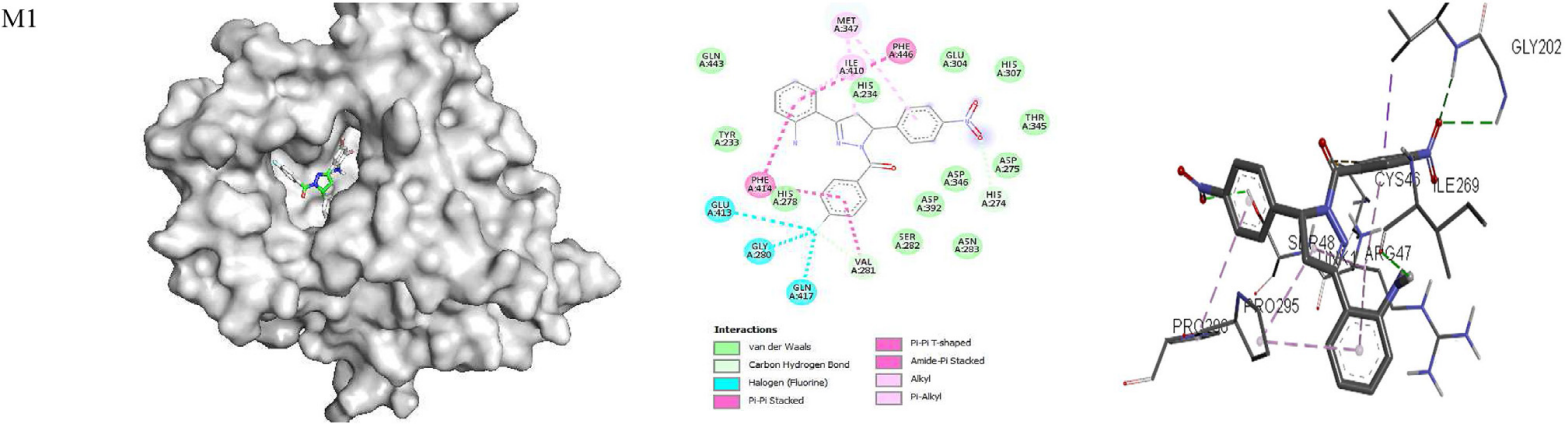

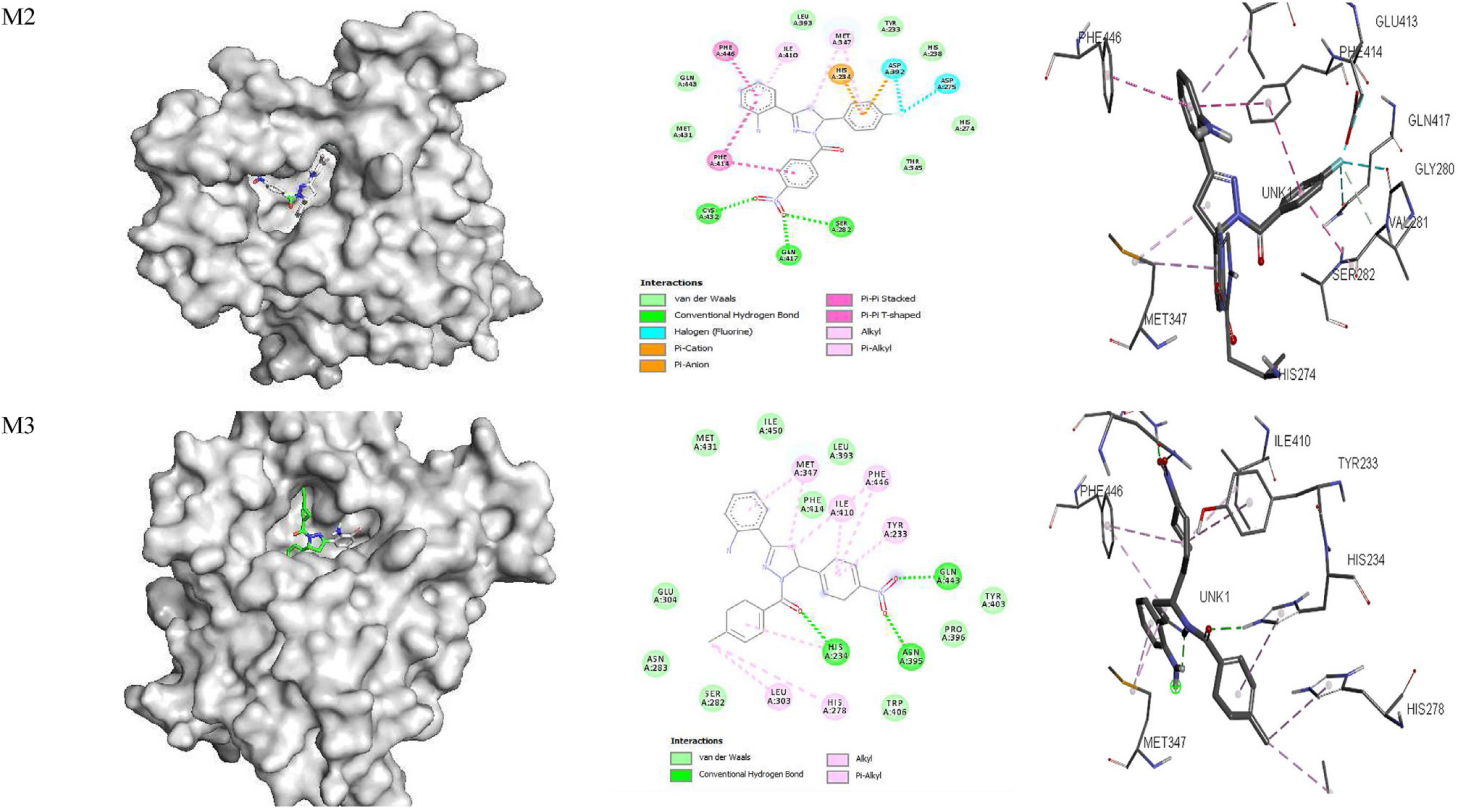

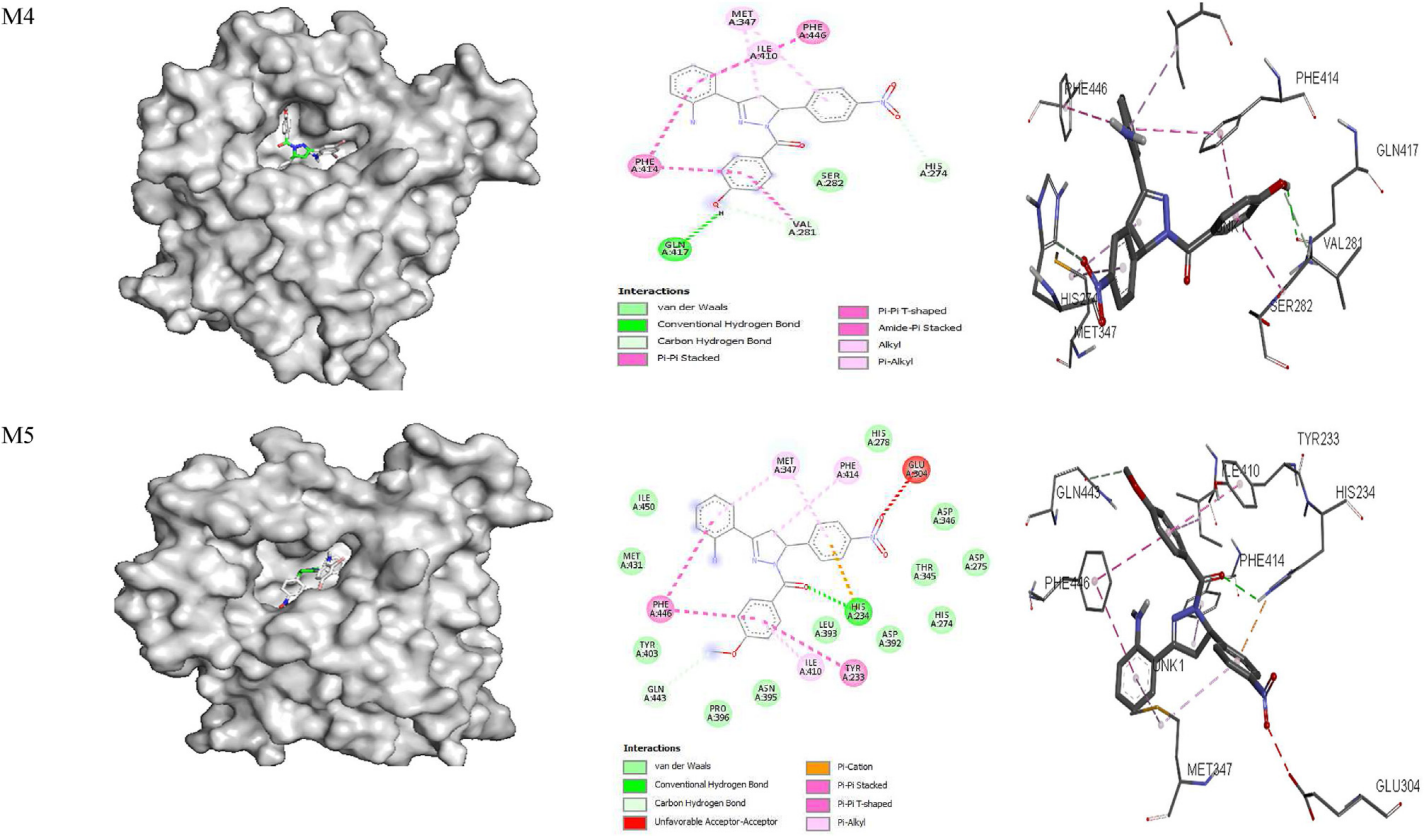

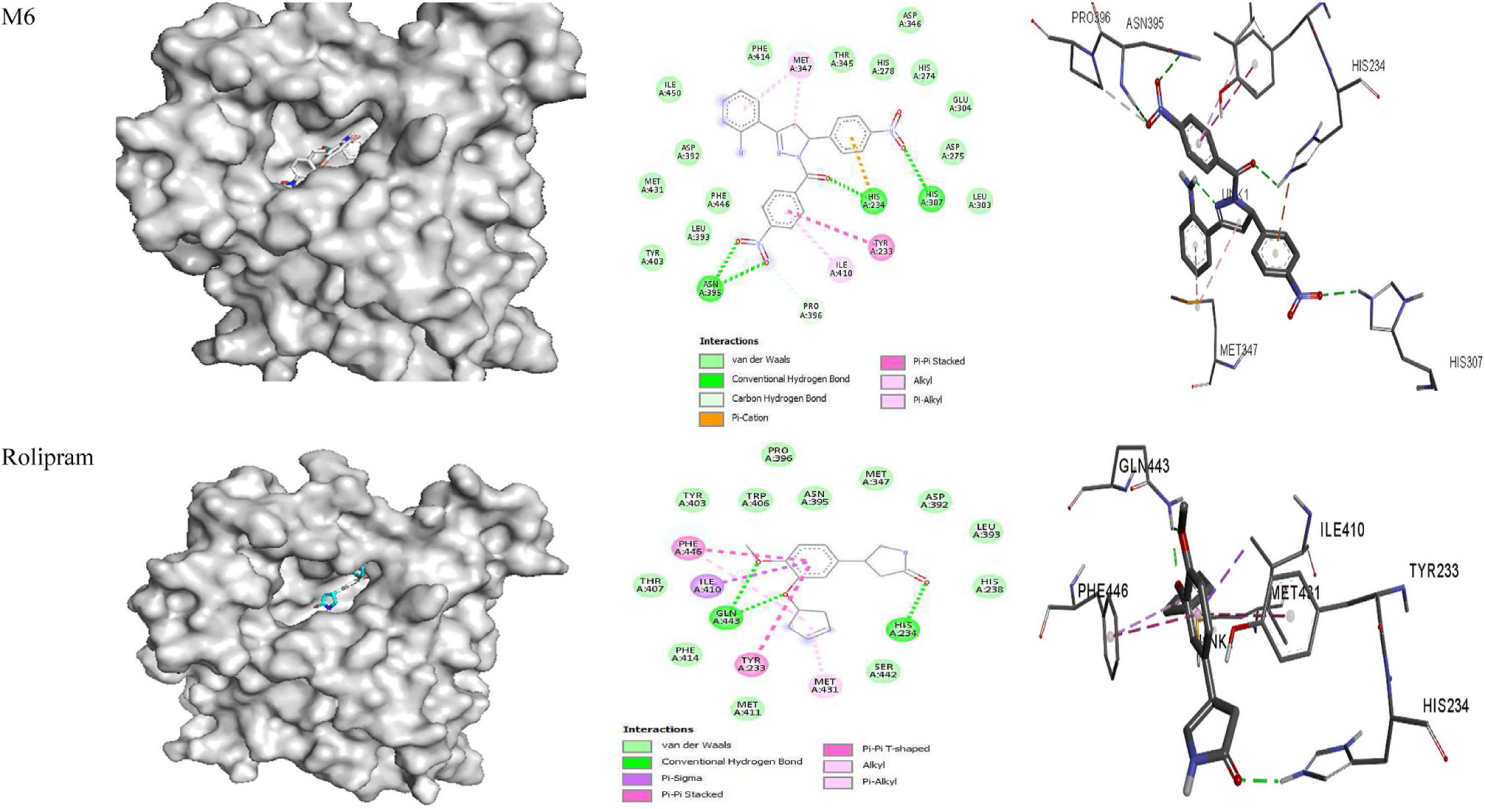
Docked complexes of the **1RO6** receptor with compounds **M1–M6**, showing interactions in the binding pocket.

The ligand bound at the active site of the **1RO6** receptor (Figure 4) revealed that MET 347 and ILE 410 are involved in alkyl-π interactions; PHE 446, PHE 414 and VAL 281 form π-π stacking, amide-π stacking and π-π T-stacking interactions; and GLU 413, GLY 280 and GLN 417 form fluorine-π interactions with **M1**. Amino acid residues PHE 446 and PHE 414 are involved in π-π stacking and form π-π T-stacking interactions; MET 347 and ILE 310 are involved in alkyl-π interactions; CYS 432, SER 282 and GLN 417 participate in hydrogen bonding; ASP 392 and ASP 275 form fluorine-π interactions; and HIS 234 and ASP 392 are involved in π-cationic and anionic interactions with **M2**. In addition, MET 347, PHE 446, ILE 410, TYR 233, LEU 303, HIS 278 and HIS 234 all form alkyl-π interactions, and ASN 395, HIS 234 and GLN 443 are involved in hydrogen bond interactions with **M3**. Amino acids PHE 414, VAL 281 and PHE 446 are involved in amide-π and π-π T-stacking interactions; GLN 417 forms hydrogen bond interactions; MET 347 forms alkyl-π interactions; and VAL 281 is involved in carbon hydrogen bond interactions with **M4**. MET 347, PHE 414 and ILE 410 form alkyl- π interactions; PHE 446 and TYR 233 are involved in π-π T-stacking and π-π T-stacking interactions; and HIS 234 forms hydrogen bond and π-cationic interactions with **M5**. Moreover, MET 347 and ILE 410 form alkyl-π interactions; ASN 395, HIS 234 and HIS 307 are involved in hydrogen bond interactions; TYR 233 forms π-π stacking interactions; and HIS 234 is involved in π-cationic interactions with **M6.** Rolipram is involved in π-π and π-π T-stacking interactions with PHE 446 and TYR 233; hydrogen bonding with GLN 443 and HIS 234; π-sigma interactions with ILE 410; and alkyl-π interactions with PHE 446 and MET 431 (Table 8 and Figure 4). The free energy for binding of rolipram with PDE (**PDB ID:** **1RO6**) in the active pocket was −9.7, −9.2, −9.4, −9.9, −8.5 and −8.9 kcal/mol for **M1–M6,** respectively, whereas −8.8 kcal/mol was calculated for rolipram. These findings suggest that all model compounds except **M5** exhibit PDE inhibitory activity than rolipram drug.

## Conclusion

DFT and molecular docking methods were performed on a set of (4-fluorophenyl)[5-(4-nitrophenyl)-3-phenyl-4,5-dihydro-1*H*-pyrazol-1-yl]methanone derivatives to calculate their molecular properties such as global electrophilicity, electron donating and accepting power, electrostatic charge distribution, and anti-hypertensive and antioxidant activity. The calculated ω^+^ revealed that the electrophilic nature of the compounds was increased by the presence of electron withdrawing groups, and the effect was particularly pronounced in the compound with two NO_2_ groups, **M6**. MEP analysis showed that amide and nitro groups on the compounds were centers of electrophilic attacks, and the magnitude of the molecular hyperpolarizability suggested that the compounds might have NLO properties.

The binding affinity for the protein-ligand complexes ranged from −8.8 to −9.3 kcal/mol—values higher than that of taurine, an antioxidant involved in inhibition of dehydrogenase (**PDB ID:** **5ADH****)** in hepatic tissue. For PDE (**PDB:** **1RO6**), the binding affinity ranged from −8.5 to −9.9 kcal/mol, whereas that of rolipram, a selective inhibitor of PDE4, was −8.8 kcal/mol, thereby indicating that only **M5** (−8.5 kcal/mol) had a lower binding affinity than rolipram. Thus, the docking results showed that these compounds may be excellent antioxidant and anti-inflammatory agents.

## Source of funding

This research did not receive any specific grant from funding agencies in the public, commercial, or not-for-profit sectors.

## Conflict of interest

The authors have no conflict of interest to declare.

## Ethical approval

Not applicable.

## Authors contributions

SB, OOA, OAK: Conceptualization; OAK, IAO, OTE, AMD: Methodology; IAO, SB, OTE, LDF, OAK, OAD, AIO, AMD and OOA: Writing- Original draft preparation; OAK and AMD: Software and Visualization; OAD and AIO: Data curation; IAO, SB, OTE, LDF, OAK, OAD, AIO, AMD and OOA: Reviewing and Editing; SB, OOA: Supervision: SB: Validation: OAK. All authors have critically reviewed and approved the final draft and are responsible for the content and similarity index of the manuscript.
